# Stability of Spiked *Chlamydia Trachomatis* and *Neisseria Gonorrhea* in Urine and Swab Specimens After Prolonged Storage at Room and Freezer Temperatures Using Aptima Combo-2 Test

**DOI:** 10.1093/ofid/ofaf388

**Published:** 2025-07-01

**Authors:** Helen Cristina Stankiewicz Karita, Amalia S Magaret, Stacy Selke, Rushlenne Pascual, Bianca Irimia, Lindley A Barbee, Anna Wald, Olusegun O Soge

**Affiliations:** Department of Medicine, University of California San Francisco, San Francisco, California, USA; Department of Pediatrics, University of Washington, Seattle, Washington, USA; Department of Biostatistics, University of Washington, Seattle, Washington, USA; Department of Laboratory Medicine and Pathology, University of Washington, Seattle, Washington, USA; Department of Global Health, University of Washington, Seattle, Washington, USA; Department of Global Health, University of Washington, Seattle, Washington, USA; Department of Medicine, University of Washington, Seattle, Washington, USA; Department of Laboratory Medicine and Pathology, University of Washington, Seattle, Washington, USA; Department of Medicine, University of Washington, Seattle, Washington, USA; Department of Epidemiology, University of Washington, Seattle, Washington, USA; Vaccine and Infectious Disease Division, Fred Hutchinson Cancer Center, Seattle, Washington, USA; Department of Laboratory Medicine and Pathology, University of Washington, Seattle, Washington, USA; Department of Global Health, University of Washington, Seattle, Washington, USA; Department of Medicine, University of Washington, Seattle, Washington, USA

**Keywords:** Aptima combo-2 test, chlamydia trachomatis, NAAT, neisseria gonorrhea, prolonged storage

## Abstract

We evaluated whether prolonged storage at room and freezer temperatures affects detection of *Chlamydia trachomatis* and *Neisseria gonorrhoeae* (CT/GC) using Aptima Combo-2 assay for research studies. Three hundred specimens were spiked with CT/GC; half were stored at room temperature and half at −80°C. All specimens remained CT/GC positive for 36 months.

Nucleic acid amplification testing (NAAT) is the recommended method for detecting *Chlamydia trachomatis* (CT) and *Neisseria gonorrhoeae* (GC) infections. It is also an essential tool for research studies including CT and GC (CT/GC) epidemiological and natural history studies, and molecular surveillance of GC antimicrobial resistance [1]. NAAT enables sensitive and specific diagnosis of CT/GC by amplifying DNA or RNA sequences using polymerase chain reaction, strand displacement amplification, or transcription-mediated amplification [2]. Several specimen types, including anogenital and throat swabs and urine specimens, can be tested for CT/GC detection by NAAT. However, the types of specimens and methods of collection and storage vary by assay manufacturer.

The Aptima Combo 2 Assay (AC2, Hologic Inc.) is a Food and Drug Administration-approved NAAT designed to detect both CT/GC in a single specimen obtained from throat, vaginal, endocervical, urethral, and rectal swabs or urine specimens [2–4]. The manufacturer recommends that specimens can be stored for 30 days (urine) to 60 days (endocervical, vaginal, urethral) at 2°C to 30°C and for 12 months at −20°C to −70°C in Aptima transport medium [4]. Studies evaluating nonstandard storage conditions are limited. One study testing spiked specimens with CT/GC stored for up to 84 days at temperatures as high as 36°C demonstrated stable detection of both pathogens [5]. Furthermore, another study showed that CT could be detected by NAAT from vaginal swabs stored up to 20 years at −80°C [6].

The increasing number of specimens generated in research studies requires freezer space, maintenance expenses, and energy utilization. Freezer storage of biological specimens obtained at remote field sites and in low-resource settings creates additional challenges, such as operating and shipping costs and risks of compromising specimen integrity due to power outages. To evaluate the impact of temperature and prolonged storage on the detection stability of CT/GC in samples collected from research studies using the AC2 assay, we analyzed throat, urine, and anogenital swab specimens spiked with CT/GC and stored them at room temperature and in a freezer for 36 months.

## METHODS

Between December 2016 and January 2017, we recruited asymptomatic volunteers without sexually transmitted infection (STI) history at the University of Washington Virology Research Clinic in Seattle, Washington. Participants self-collected anogenital and throat swabs in duplicate using the Aptima Multitest Swab Specimen Collection Kit [7]. We collected urine in a sterile, preservative-free container and processed it by adding 2 mL of urine to the Aptima transport media in duplicate [8]. The University of Washington institutional review board approved this study, and volunteers provided verbal consent.

We cultured and quantified the infectious units per milliliter for CT and the colony-forming units per milliliter for GC, using previously established protocols [9–13]. We spiked each participant's specimen with cultured CT/GC strains to obtain a final concentration of 10^4^ infectious units/mL of CT (L2/434) and 5 × 10^5^ colony-forming units/mL of GC (CDC-F18/ATCC 49226) based on the previously reported average CT [14, 15] and GC [16, 17] concentrations. Each Aptima tube had sufficient volume for testing at 3 timepoints (0, 12, and 24 or 36 months). After spiking the specimens, we tested them with AC2 using the Panther System and confirmed CT/GC detection (month 0). We stored spiked specimens at room temperature and −80°C in duplicate; each frozen specimen was subjected to 2 freeze-thaws at the time of AC2 testing. At 12 months of storage, we retested all specimens at room temperature and freezer for CT/GC. After 24 months of storage, we selected half of the specimens from each group using simple randomization and retested them for CT/GC. We them reevaluated the residual specimens from each group at 36 months of storage. The AC2 software automatically reported individual CT and GC test results as positive, negative, equivocal, or invalid, based on the manufacturer-defined relative light unit (RLU) thresholds (see Supplementary Appendix). We compared qualitative (positive/negative) and semiquantitative value (RLU) results at baseline with those at 12, 24, and 36 months [4].

## RESULTS

**Figure 1.**
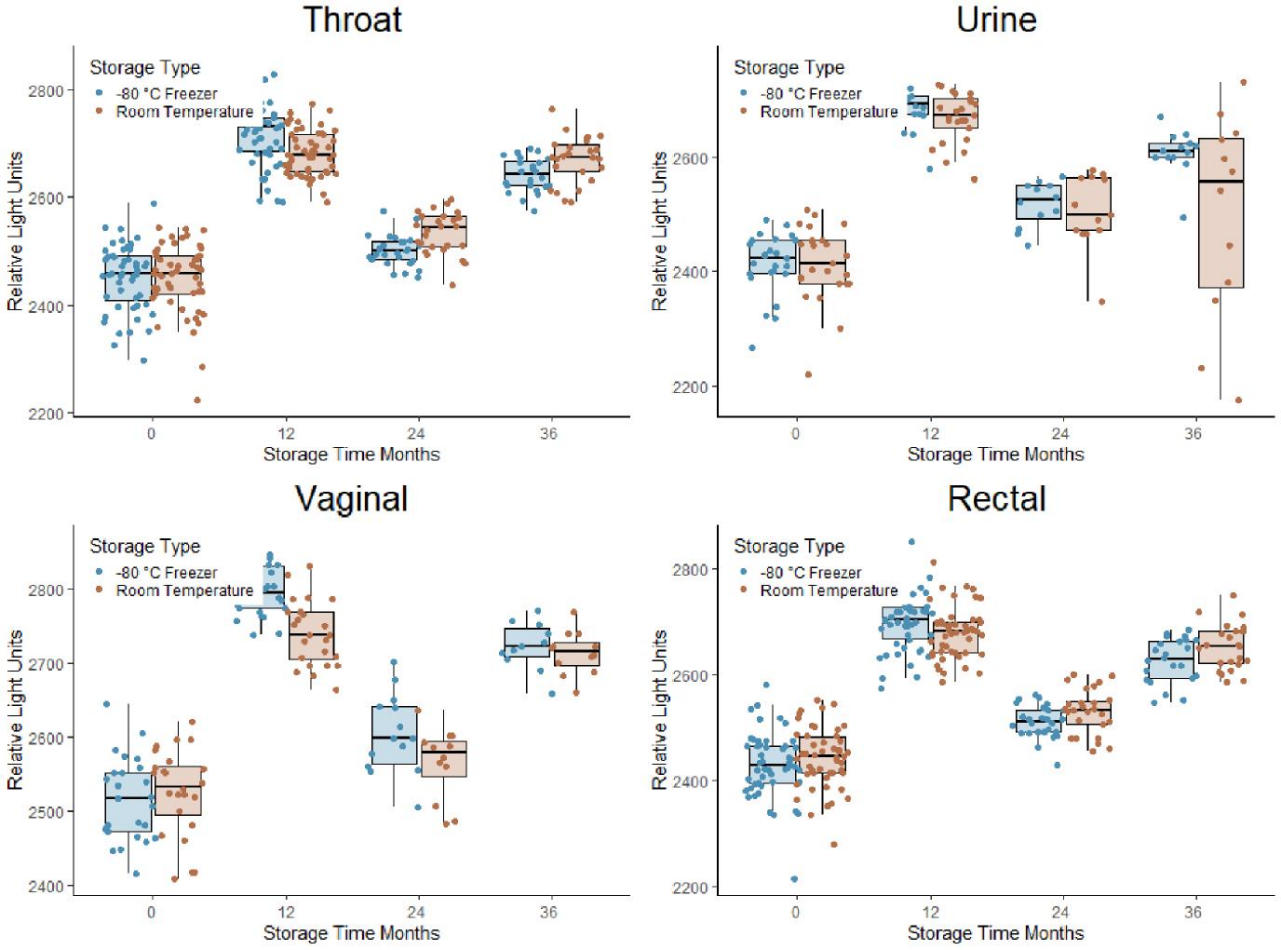
Comparison of *Chlamydia trachomatis* (CT) and *Neisseria gonorrhoeae* (GC) relative light units in throat, vaginal, and rectal swabs and urine specimens stored in a −80°C freezer and at room temperature over the 36-month study period. Each dot represents a single specimen spiked with CT and GC.

We collected 300 specimens; women provided 50 throat, 50 vaginal, and 50 rectal swabs, and men provided 50 urine, 50 throat, and 50 rectal swabs. Immediately after spiking, we confirmed the detection of CT/GC in all clinical specimens, with RLU values comparable to those of clinical specimens from patients co-infected with CT/GC (Figure 1). Both room temperature and freezer specimens (n = 150 each) consistently tested positive for CT/GC at 12, 24, and 36 months of storage. Over the first year of freezer storage, median changes in RLU ranged from +270 to +280, depending on specimen type, and they ranged from +190 to +260 among room temperature samples. Semiquantitative changes were comparable according to storage type at years 2 and 3 (Figure 1). However, within each year and specimen type, the median differences in RLU values by storage method (freezer minus room temperature) were smaller and centered around zero, ranging from −36 to +40.

## DISCUSSION

Our findings indicate that throat, vaginal, and rectal swabs and urine specimens processed in the Aptima transport medium containing spiked CT/GC remain positive with great accuracy over an extended storage period, both frozen and at room temperature, suggesting stability of the specimens over a range of temperatures including ambient temperature, refrigeration (2–8°C), freezer (−20°C), ultra-low freezer (−80°C). RLU values fluctuated slightly over 3 years possibly from lot-to-lot variations in AC2 reagents, varying reagent processing efficiency by different laboratory staff, freezing-thawing cycles that could impair subsequent release of the cellular DNase, differences in degrading enzymes or inhibitory substances, or concentration of CT/GC targets over time from decrease in specimen volume [18, 19]. However, consistent qualitative detection in all specimens demonstrated that room temperature storage conditions did not affect CT/GC detection by AC2.

Building on previous studies that demonstrated the stability of CT/GC detection beyond the manufacturer specifications, our study further investigated the stability of the AC2 assay. Rose et al. evaluated 20 vaginal swabs positive for CT stored for 20 years and found that 18 (90%) of the specimens remained positive upon reanalysis using ribosomal RNA-based transcription-mediated amplification [6]. Another study evaluated the Aptima assay on dry CT-seeded swabs after storage at 4°C, 23°C, or 36°C for up to 84 days, observing no significant impact from prolonged storage or temperature variation [5]. They also tested swabs seeded with CT/GC that were mailed and exposed to temperatures exceeding 30°C, with no evidence of signal degradation, even for samples that took more than 5 weeks to reach the laboratory [6]. In our study, we extended these findings by demonstrating that throat, urine, vaginal, and rectal specimens spiked with CT/GC remain stable at room temperature and in the freezer for up to 36 months. Although specimens spiked with known concentrations of cultured organisms are generally acceptable for validation and evaluation of the performance of molecular assays [20, 21], they do not reflect the full complexity of real-world clinical specimens, including pathogen diversity and the biological variations found in natural infections [18]. Therefore, given that our study was conducted within the context of research, we do not advocate off-label storage of AC2 specimens intended for clinical use.

This study has several limitations. First, our results are limited by the slight variation in room temperature between 21°C to 23°C. The controlled laboratory room temperature at the University of Washington, Seattle, was lower than in tropical/subtropical settings, where a higher ambient temperature or humidity could decrease the specimen stability. Second, we did not test the specimens for CT/GC before spiking. However, specimens were collected from asymptomatic volunteers without history of STIs similar to other asymptomatic patients enrolled in other studies in our research clinic who consistently tested negative for CT/GC by AC2. Moreover, we conducted testing immediately after spiking to confirm detection, and the RLU values were comparable to those observed in CT/GC-positive clinical specimens. Third, we used a single analyzer for the simultaneous detection of CT/GC, which may impact the reproducibility of our findings compared to other molecular detection platforms that may be more widely available globally. Additionally, all specimens were collected using the Hologic transport medium, which could limit the generalizability of our findings to resource-limited settings where generic preservative medium may be used. Fourth, we did not separately test the stability of specimens spiked either with CT or GC alone. However, the observed RLU values were suggestive of robust detection of CT/GC, and most commercial NAAT assays concurrently detect CT/GC without compromising the efficacy of detecting single infection with CT or GC. Furthermore, each specimen was spiked with standardized concentrations of CT/GC, calculated to approximate values of clinical specimens, with no specimens spiked at different concentrations or actual clinical specimens included [14–17]. Similar to other pathogens, such as herpes simplex virus, specimens with a low CT/GC quantity may degrade faster during room temperature storage, limiting detection [22]. Therefore, the findings in our study should not be generalized or directly extrapolated to clinical samples or settings. These findings could be extended further by spiking specimens with varying cell densities of CT/GC (low/medium/high) or by using specimens from infected patients to reflect the variability observed among clinical specimens.

Ensuring the stability of CT/GC during prolonged storage at room temperature could significantly lower STI research costs. This approach could also facilitate epidemiological and natural history studies.

## Supplementary Material

ofaf388_Supplementary_Data

## Supplementary Data

Supplementary materials are available at *Open Forum Infectious Diseases* online. Consisting of data provided by the authors to benefit the reader, the posted materials are not copyedited and are the sole responsibility of the authors, so questions or comments should be addressed to the corresponding author.
