# Supplementary material for: Stability of Spiked Chlamydia Trachomatis and Neisseria Gonorrhea in Urine and Swab Specimens After Prolonged Storage at Room and Freezer Temperatures Using Aptima Combo-2 Test
Source: Open Forum Infect Dis. 2025 Jul 1;12(7):ofaf388. doi: 10.1093/ofid/ofaf388 (PMC12282393; doi:10.1093/ofid/ofaf388)
Supplement: ofaf388_Supplementary_Data [file ofaf388_supplementary_data.docx]

**Stability of Spiked *Chlamydia trachomatis* and *Neisseria gonorrhea* in Urine and Swab Specimens after Prolonged Storage at Room and Freezer Temperatures Using Aptima Combo-2 Test.**

**Supplementary Appendix**

Table of Contents

[Summary of Aptima Combo2 Assay principles of the procedure^1^ 3](#_Toc190520810)

[Table S1. Aptima Combo 2 test interpretation according to the manufacturer’s package insert^1^ 4](#_Toc190520811)

[Reference 5](#_Toc190520812)

# Summary of Aptima Combo2 Assay principles of the procedure^1^

The Aptima Combo® 2 Assay detects ribosomal RNA (rRNA) from Chlamydia trachomatis (CT) and/or Neisseria gonorrhoeae (GC) using nucleic acid amplification and target capture technology. This test is compatible with the Tigris DTS® System, Panther System, or semi-automated DTS Systems. Per the manufacturer’s recommendations, specimens are collected and placed in an Aptima transport medium that releases rRNA targets and protects them from degradation. The rRNA is isolated using capture oligomers attached to magnetic microparticles, which bind to specific target regions, and the capture complex is then isolated by reducing the reaction temperature, enabling hybridization with poly-deoxythymidine molecules on the particles. The amplification is achieved using specific primers for CT 23S rRNA and GC 16S rRNA. The detection is done through chemiluminescent hybridization of the amplified products. Labeled DNA probes, specific to each target amplicon, form stable RNA: DNA hybrids, and the system detects the signal as Relative Light Units (RLU) in a luminometer. The Relative Light Units (RLU) quantify the intensity of chemiluminescent signals, with assay results determined by a cut-off based on the total RLU and the kinetic curve type. Notably, RLU values can vary based on the assay used, specimen type, and laboratory protocols. Therefore, interpreting RLU values requires adherence to the specific guidelines provided by the assay manufacturer and consideration of the clinical context. In clinical practice, RLU values are interpreted within specific thresholds to determine the presence of infection.

| **Table S1.** Aptima Combo 2 test interpretation according to the manufacturer’s package insert^1^ | | | |
| --- | --- | --- | --- |
| **Kinetic Type** | **Total RLU (x1000) to give CT Result** | | |
|  | Negative | Equivocal | Positive |
| CT only | 1 to < 25 | 25 to < 100 | 100 to < 4,500 |
| CT and GC | 1 to < 85 | 85 to < 250 | 250 to < 4,500 |
| CT Indeterminate | 1 to < 85 | 85 to < 4,500 | N/A |
|  | | | |
| **Kinetic Type** | **Total RLU (x 1000) to give GC Result** | | |
|  | Negative | Equivocal | Positive |
| GC only | 1 to <60 | 60 to < 150 | 150 to < 4,500 |
| CG and CT | 1 to < 85 | 85 to < 250 | 250 to < 4,500 |
| GC indeterminate | 1 to < 85 | 85 to < 4,500 | N/A |

# Reference

1. https://www.hologic.com/sites/default/files/package-insert/502446-IFU-PI_003_01.pdf. Aptima Combo 2® Assay (Panther®  System).
